# On-Chip Magnetic Bead Manipulation and Detection Using a Magnetoresistive Sensor-Based Micro-Chip: Design Considerations and Experimental Characterization

**DOI:** 10.3390/s16091369

**Published:** 2016-08-26

**Authors:** Chinthaka P. Gooneratne, Rimantas Kodzius, Fuquan Li, Ian G. Foulds, Jürgen Kosel

**Affiliations:** Computer, Electrical and Mathematical Sciences and Engineering, King Abdullah University of Science and Technology, Thuwal 23955, Saudi Arabia; rimantas.kodzius@kaust.edu.sa (R.K.); fuquan.li@kaust.edu.sa (F.L.); ian.foulds@ubc.ca (I.G.F.); jurgen.kosel@kaust.edu.sa (J.K.)

**Keywords:** magnetoresistive sensor, superparamagnetic beads, micro-chip, micro-actuator, nanoparticles, magnetophoresis, magnetic detection, numerical analysis, lab-on-a-chip, microfabrication

## Abstract

The remarkable advantages micro-chip platforms offer over cumbersome, time-consuming equipment currently in use for bio-analysis are well documented. In this research, a micro-chip that includes a unique magnetic actuator (MA) for the manipulation of superparamagnetic beads (SPBs), and a magnetoresistive sensor for the detection of SPBs is presented. A design methodology, which takes into account the magnetic volume of SPBs, diffusion and heat transfer phenomena, is presented with the aid of numerical analysis to optimize the parameters of the MA. The MA was employed as a magnetic flux generator and experimental analysis with commercially available COMPEL™ and Dynabeads^®^ demonstrated the ability of the MA to precisely transport a small number of SPBs over long distances and concentrate SPBs to a sensing site for detection. Moreover, the velocities of COMPEL™ and Dynabead^®^ SPBs were correlated to their magnetic volumes and were in good agreement with numerical model predictions. We found that 2.8 μm Dynabeads^®^ travel faster, and can be attracted to a magnetic source from a longer distance, than 6.2 μm COMPEL™ beads at magnetic flux magnitudes of less than 10 mT. The micro-chip system could easily be integrated with electronic circuitry and microfluidic functions, paving the way for an on-chip biomolecule quantification device.

## 1. Introduction

Superparamagnetic beads (SPBs) have attracted a lot of interest for sorting, separating, purifying and detecting biomolecules in assays [1,2,3,4,5,6,7,8,9,10,11,12]. A large number of magnetic micro-devices have been developed in recent times for on-chip manipulation of SPBs [3,13,14,15]. In these devices, the magnetic force used to manipulate SPBs is provided by micrometer sized electromagnets, soft-ferromagnetic structures, or some combination of both. However, some of these do not allow precise and controlled SPB manipulation, resulting in low trapping efficiencies, while some are not designed for integration with magnetic sensors for detection of SPBs. More recently, domain wall motion in nanometer thick permalloy patterns has been used to precisely transport SPBs to a target location [16,17] as well for detection of SPBs [18,19,20,21,22]. However, these devices employ bulky off-chip electromagnets or permanent magnets as magnetic field generators, which must be aligned to features on the micro-device. Moreover, the fabrication is more complex and time consuming compared to simple micro-electromagnets or permanent magnets. However, one of the main disadvantages of on-chip electromagnets compared with permanent magnets and external magnetic field generators is that they can increase the temperature of the chip when there is current flowing through them. An increase in the temperature of the solution might alter the SPBs’ behavior and/or the biomolecules attached to it. However, how much of an influence this has on the results depends on the material the electromagnet is made of and its dimensions, as well as the thermal time constants of the experimental solution. Also, the duration of current flow through an electromagnet is an important parameter to consider in the design of on-chip electromagnets.

A significant amount of work has been conducted on utilizing magnetoresistive (MR) sensors to detect SPBs [23,24,25,26,27,28]. MR sensors are highly sensitive, inexpensive, and compatible with most lab-on-a-chip applications. MR sensor-based chips, which can also be combined with magnetic microstructures, have many potential biomedical applications, such as in the quantifications of protein interactions [29,30], DNA analysis [31,32,33,34], detection of pathogens [35,36], cancer [37,38,39] and HIV [40], as well as in the diagnosis of myocardial infarction [41] and sepsis [42]. An excellent overview of how to design and fabricate MR sensor-based chips can be found in [43]. The research groups of Freitas [24,31,32,44], Borghs [44,45,46] and Prins [34,47,48] were the pioneers of integrating magnetic microstructures and MR sensors on-chip to manipulate and detect SPBs. They used tapered current lines to manipulate low concentrations of SPBs towards MR sensors for detection. However, SPBs that are far away from the sensor might take a long time to be manipulated to the sensing area utilizing the tapered current line approach. Moreover, tapered current lines do not and/or are unable to cover a large area in the spatial *x*-*y* range, and manipulation was performed on a high concentration of SPBs without precise control of them. Therefore, when we first started working on the manipulation of SPBs the aim was to build a simple, inexpensive and practical micro-device that could manipulate SPBs in a controlled manner, and also be integrated seamlessly with an MR sensor. Our initial work successfully demonstrated that by switching current between conducting lines, SPBs could be moved to a sensing zone for detection [49,50,51]. It has also been used recently to analyze the magnetophoretic velocity of SPBs [52,53]. In [54,55] we presented integrated micro-chips for rapid detection of SPBs and provided proof-of-concept experimental results. Yet, in these devices current was applied to only one loop at a time. This meant that SPBs in the vicinity of loops without an applied current were sometimes lost due to diffusion caused by thermal energy. Therefore, a high concentration of SPBs was used in these experiments to demonstrate the concept of controlled manipulation and detection of SPBs.

In this paper we present a uniquely designed magnetic actuator (MA) that produces a travelling magnetic field. This ensures all SPBs on the MA are eventually manipulated to the sensing site for detection. The MA is made of conducting loops that carry an electric current in order to manipulate SPBs. However, the conducting loops are connected in sets, and current is sequentially applied to one set at a time to increase the probability that all SPBs on the MA will be transported to a target location. We also describe a design framework for the optimization of the MA by numerical analysis, taking into account not only the diffusion of SPBs, but also factors such as the magnetic volume of the SPBs based on the magnetite/iron oxide content of the SPB, volume susceptibilities at working fields instead of saturation fields, since the susceptibilities at low magnetization fields are higher than susceptibilities at magnetizations approaching saturation, and heat transfer phenomena due to Joule heating. Experiments are performed to verify the numerical analysis and demonstrate the manipulation of bare and tagged SPBs using the MA, and the integration of the MA to a magnetoresistive sensor, to form a fully integrated micro-chip for the detection of bare SPBs.

## 2. Theoretical Basis

An SPB dispersed in a liquid medium under the influence of a magnetic flux density will experience a magnetic force ***F_m_*** defined by [3,13]:

(1) $$F_{m}=\frac{V_{mag}\Delta \chi }{\mu_{0}}\left(B\cdot \nabla \right)B$$

where *V_mag_* (m^3^) is the volume of magnetic material in an SPB, Δ*χ* is the difference in volume susceptibility between the SPB and the surrounding liquid medium, *μ*_0_ = 4π × 10^−7^ (Vs/Am) is the permeability of free space and ***B*** (T) is magnetic flux density. Δ*χ* in a water medium is equal to the *χ* of the SPB, since *χ* of water is <<*χ* of an SPB.

The drag force, the most significant force that opposes the manipulation of SPBs, is:

(2) $$F_{d}=-6\pi \eta rv$$

where *η* (kg/sm) is the viscosity of the fluid medium, *r* (m) is the hydrodynamic radius of the SPB and ***v*** (m/s) is the velocity.

The diffusion of an SPB over a distance *L* can be calculated as:

(3) $$L=\sqrt{2Dt}$$

where *D* = *k_B_T*/*f_d_*; *k_B_* is the Boltzmann constant, *T* (K) is the temperature, *f_d_* = 6*πηr* is the drag coefficient and *t* (s) is the characteristic measurement time.

The majority of works in the literature did not consider the optimization of the manipulating structure by taking into account diffusion of the particles, but rather focused on the manipulation and detection of either bare SPBs or biomolecules tagged with SPBs, mostly in a flow.

We define the ratio of the distance an SPB travels due to diffusion to the distance an SPB travels due to the magnetic force as the error caused by diffusion, when a magnetic force is used to attract an SPB from a certain distance:

(4) $$e=\frac{L}{v_{x}t}$$

where *v_x_* = *F_m,x_*/*f_d_* and *F_m,x_* (N) is the magnetic force in the *x*-direction.

The equation shows that the smaller the magnetic force, the higher the error caused by diffusion, and the lower the chance of an SPB being attracted towards the magnetic source. The equation also only considers movement of SPBs in one direction, the *x*-direction, from the edge of one conducting loop to another, since generally the majority of SPBs are attracted towards the surface of the MA due to gravity. The sedimentation time also increases in the presence of a magnetic force. Moreover, there is no bulk flow of the sample solution containing SPBs and biomolecules, so the effect of advection is not taken into account. Therefore, *e* is used as a design criterion to optimize the spacing between the conducting loops.

**Figure 1:**
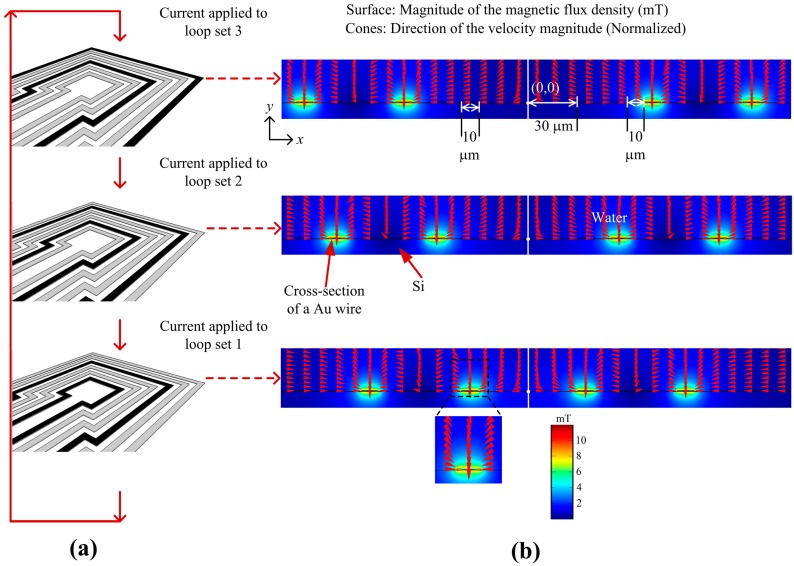
(**a**) Design of the magnetic actuator; (**b**) Magnitude of magnetic flux density (surface plot) when 125 mA of electric current was applied sequentially to the 3 loop sets.

**Figure A1:**
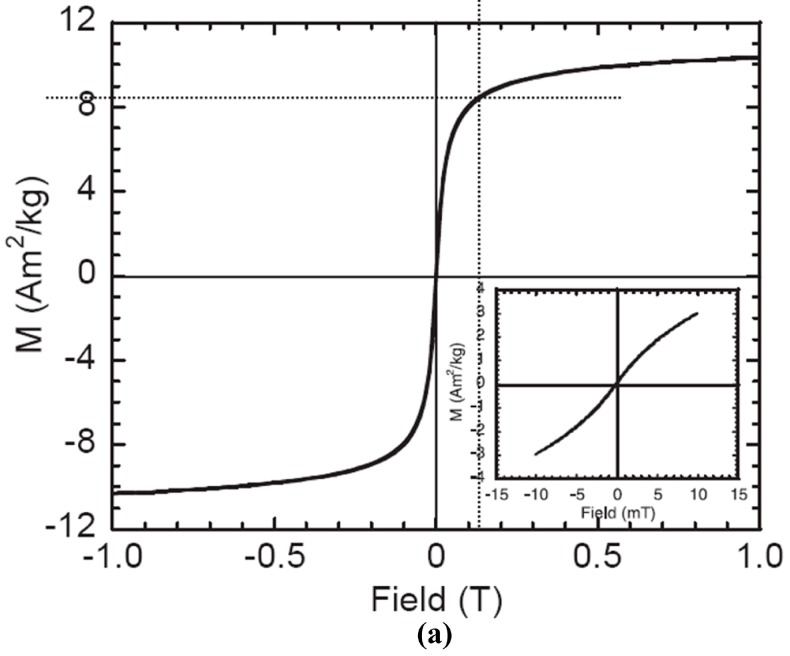

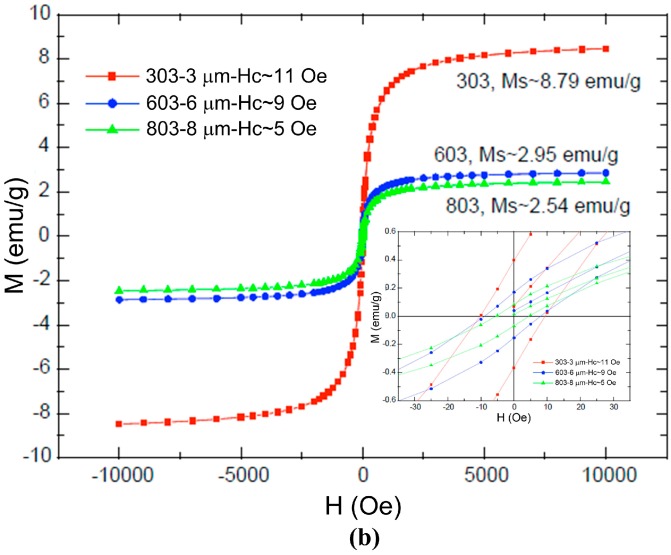
Magnetization curves for (**a**) M-270 streptavidin beads and (**b**) COMPEL™ beads. (Reprinted with permission from refs. [56]—Copyright 2005 Elsevier, and [57]—Copyright Bangs Laboratories, Inc.™).

The MA consists of square-shaped micro-loops as shown in Figure 1a. Applying current to a loop produces a magnetic flux with a gradient and therefore any SPB in the vicinity of the loop will be magnetized and attracted to the loop. At the start of the manipulation process current was applied to loop set 3 and the SPBs will be attracted to any loop in set 3 as shown in Figure 1a. Then, by applying current sequentially to loop sets 2 and 1 the SPBs can be transported towards the innermost loop. By repeating this procedure all SPBs on the MA can be transported to the target location. Numerical analysis was performed with commercial finite-element software (COMSOL^®^) on 2-D symmetric square loop models of different dimensions. Since the length of the loops was much larger than the width of the conductors, the 2-D approximation provides sufficiently accurate results, except for the corners of the loops. The spatial coordinates of the 2-D plane were *x* and *y*, and the coordinate center was located at the center of the loops at the surface of the substrate. The loops were made of Au and had a cross-sectional area of 10 µm × 0.5 µm. As shown in Figure 1b, the innermost square loop was 30 μm from the center of the MA and the edge-edge distance *d* of the loops was 10 μm. In the model, the scales in the *x* and *y* direction were 20 µm. Three current values, 100 mA, 125 mA and 150 mA, were used for simulation. In Figure 1b, a current *I* of 125 mA was applied to the loops in the *z*-direction, which, in this case, was into the loops on the left side of the symmetrical model and out the loops on the right side of the symmetrical model, in the *x-y* plane. The choice of parameters is explained in the Results and the Discussion sections. The boundary of the surrounding environment was magnetic insulation (Dirichlet condition). The model was meshed with ‘Lagrange-Quadratic’ elements, meaning that the solution was approximated with second-degree polynomials, and a static analysis was carried out. Figure 1b shows the magnitude of magnetic flux density at the edges of the loops is 10 mT, and this is within the linear range of the SPBs used in the experiments (see Figure A1). Since for a current loop the magnitude of the magnetic flux density is inversely proportional to the cube of the distance from the source, current needs to be switched between the loop sets to concentrate SPBs to the innermost loop. The cones in Figure 1b demonstrate the direction of the velocity of an SPB. The direction of the cones near the loops were towards the surface of the loops, where the magnitude of the magnetic flux density was the highest, i.e., towards increasing gradients of a magnetic flux density.

## 3. Materials and Methods

### 3.1. Fabrication of the Magnetic Actuator

**Figure 2:**
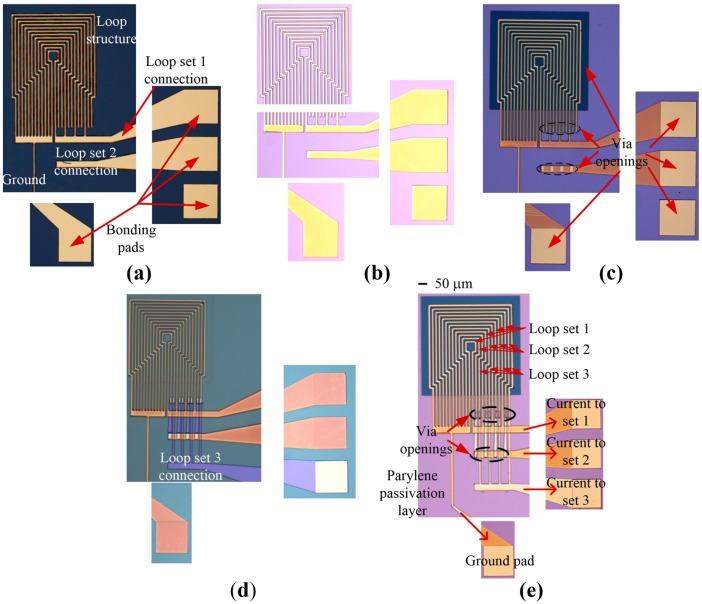
Fabrication of the micro-magnetic actuator. (**a**) Gold wires after deposition and patterning; (**b**) Parylene passivation layer on top of gold wires; (**c**) Vias and pads opened by oxygen plasma etching; (**d**) Patterned photoresist layer to connect the loop structures to bonding pads; (**e**) Final fabricated device.

Planar, square-looped structures were fabricated by photolithography, sputter deposition and etching techniques on a silicon/silicon dioxide (Si/SiO_2_) wafer (single side polished). The dimensions of the MA were the same as in the simulation model. 4 µm of positive photoresist (AZ ECI 3027) were spin coated onto a 4″ wafer (Spin Coater, Headway Research, Inc., Garland, TX, USA) and post-baked at 100 °C for 1 min. The photoresist was exposed to UV light with a constant dose of 200 mJ/cm^3^ for 3 s (EVG 6200 Contact Aligner, EV Group, Sankt Florian Am Inn, Oberosterreich, Austria). The wafer was then developed for 1 min in a tetramethylammonium hydroxide (TMAH) solution (AZ 726). Following this, 20 nm of chromium (Cr), as an adhesion layer, and 500 nm of Au were deposited onto the wafer by DC magnetron sputtering (Denton Explorer 30, Denton Vacuum, Moorestown, NJ, USA). Argon gas was used at a pressure of 4 Pa, and the DC power was set to 800 W. A lift-off process was then employed, in order to establish the patterned Au structures shown in Figure 2a. Next, the wafer was coated with a 1 µm thick layer of Parylene C, as shown in Figure 2b (Labcoater^®^ 2, Speciality Coating Systems, Indianapolis, IN, USA). This passivation layer was required to prevent a short circuit between the electrical connection of the loop sets. Plasma etching (NanoPlas DSB 6000 Boost, Indo-French High-Tech Equipments, Mumbai, Maharashtra, India), at RF power of 800 W, temperature (chamber and substrate) of 100 °C, pressure of 67 Pa and O_2_ flow rate of 100 sccm (Figure 2c), was performed to open the Parylene over the bonding pads as well as an area in the three loop sets for dispensing experimental solutions. Finally, the three loop sets were connected to the bonding pads by sputter deposition of 20 nm of Cr and 500 nm of Au followed by a lift-off step (Figure 2d). The fabricated MA is shown in Figure 2e.

### 3.2. Sample Preparation and Experimental Setup for SPB Manipulation

Two types of SPBs, COOH modified COMPEL™ beads that have a radius of 3.1 µm and a ferrite content of 5.5% and streptavidin modified Dynabeads^®^ M-270 that have a radius of 1.4 µm and a ferrite content of 14%, were employed to test the MA. These SPBs were chosen due to their excellent dispersity in de-ionized water ensuring clear visual observations. To provide a suitable SPB density, the original density of 3.691 × 10^8^ COMPEL™ beads per mL was diluted 100 times with deionized water (Dilution 1), then a 10 µL sample of Dilution 1 was diluted 20 times (Dilution 2), a 5 µL sample of Dilution 2 was further diluted 6 times (Dilution 3) and, finally, a 1 µL of Dilution 3, amounting to approximately 30 SPBs, was dispensed to the surface of the MA. The original density of 6.5 × 10^8^ Dynabeads^®^ per mL was diluted as above except for a final dilution (Dilution 3) of 11 times to obtain 30 SPBs in 1 µL of sample fluid.

**Figure A2:**
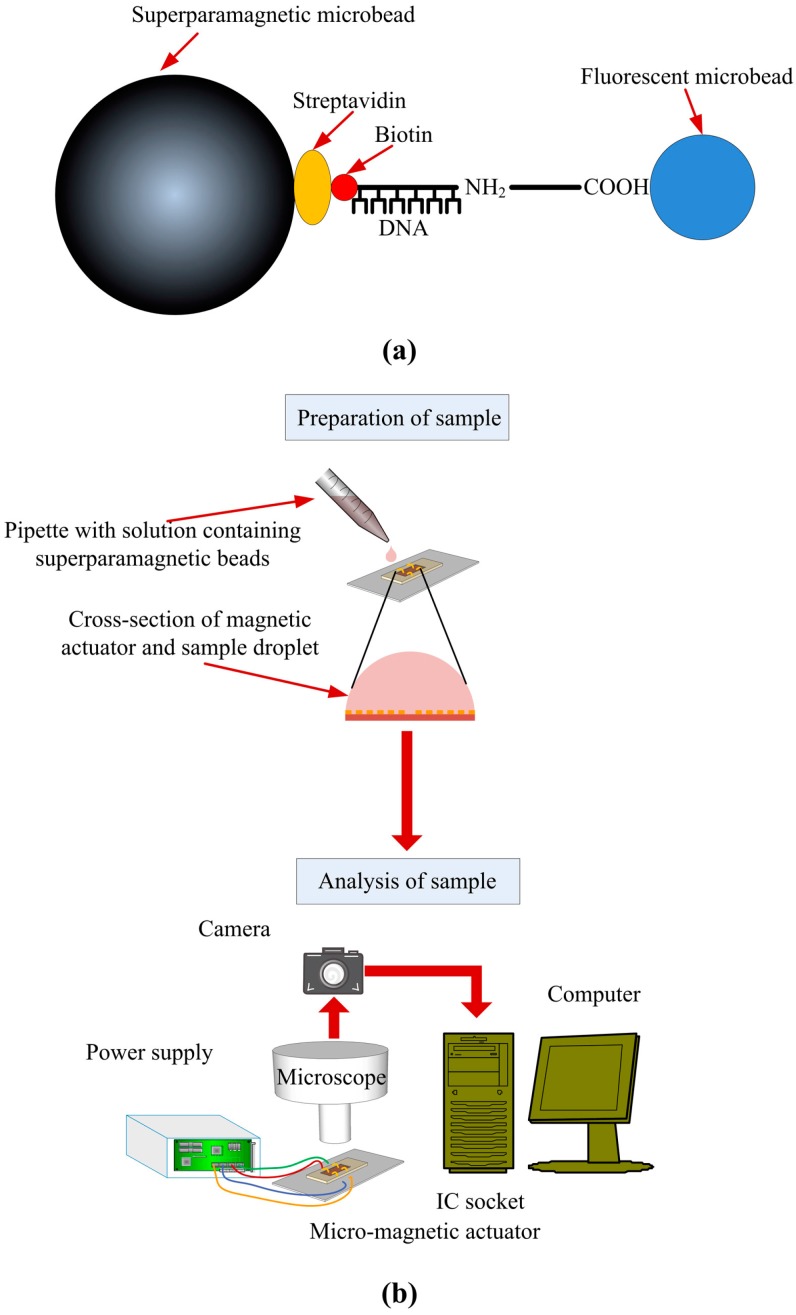
(**a**) Attachment of DNA to a superparamagnetic bead; (**b**) Experimental setup for manipulation of magnetic beads by the magnetic actuator.

In order to observe the effect binding has on the velocity of Dynabeads^®^, we tagged non-magnetic microspheres with Dynabeads^®^. The complete process to tag polystyrene particles with Dynabeads^®^ utilizing nano-sized DNA strands is as follows (See Figure A2a): 1 µm diameter Fluoresbrite^®^ YG Carboxylate Microspheres (polystyrene YG particles, PolyScience Inc., Warrington, PA, USA, Cat. No. 15702) were functionalized with oligonucleotide 23ca6_SRY93F_BIO (5′-Amino C6–ATAAGTATCGACCTCGTCGGAAG–Biotin-3′) through 1-ethyl-3-(3-dimethylaminopropyl) by carbodiimide hydrochloride (EDAC) chemistry. After functionalization the SPBs were resuspended into 1× PBS containing 0.02% sodium azide (NaN_3_) to the final concentration of 1.15 × 10^9^ particles/mL. 20 µL of the original SPBs with concentration 6.5 × 10^8^ SPBs/mL were washed and then mixed with 2 µL oligo-functionalized YG particles, 22 µL of 2× Binding and Wash solution (consisting of 10 mM Tris-HCl (pH 7.5), 1 mM EDTA and 2M NaCl: designated as 2× B&W) and incubated for 3 h at room temperature (23 °C) with continuous gentle agitation to prevent settling of the SPBs. After functionalization of the streptavidin SPBs with fluorescent particles using oligonucleotide as a bridging molecule, the SPBs were washed with 180 µL 2× B&W by placing the tube (Axygen, Corning, NY, USA, Cat. No. MCT-060-L-C Maximum recovery) containing the SPBs on a magnet for 1 min, followed by removal of the supernatant, another washing step with 180 µL of ddH_2_O and re-suspension of the SPBs into 100 µL of fresh ddH_2_O. The final solution was prepared by mixing 30 µL of conjugated beads (magnetic with fluorescent) with 10 µL of Fluoresbrite^®^ Polychromatic Red Microsphere solution (polystyrene PC particles, PolyScience Inc., Cat. No. 18660; for the background) (1 µm diameter, 0.455 × 10^10^ particles/mL) and 60 µL of ddH_2_O. As a result, the heterogeneous sample solution contained polystyrene YG particles attached to SPBs that expressed a fluorescent blue signal, and unattached, non-magnetic polystyrene PC particles that expressed a fluorescent green signal. Therefore, both could clearly be distinguished from each other, and their behavior and trajectory could be observed with fluorescence microscopy.

Experiments on trapping and manipulating SPBs were performed with the setup shown in Figure A2b. The fabricated MA chip was glued to an IC-socket, and electrical connections were established by wire bonding. A dual output DC power supply (Agilent, Santa Clara, CA, USA, E3646A) was utilized to apply current of 125 mA to the different loop sets. A high performance, optical fluorescence microscope with a manual stage (Eclipse L200N, Nikon, Tokyo, Japan), equipped with a CCD camera (Nikon Digital Sight DS-Fi1) connected to a computer was used to observe the experiments and to obtain still images. The still images were taken once the SPBs were in the vicinity or trapped at a given loop set. These images were then arranged in sequence, in order to study the movement of the SPBs due to magnetic force. The SPB manipulation efficiency was monitored for a number of cycles, to ensure repeatability, in which each cycle consisted of sequentially applying current to all three loop sets.

### 3.3. TMR Sensor Integration and Experimental Setup for Magnetic Bead Detection

**Figure 3:**
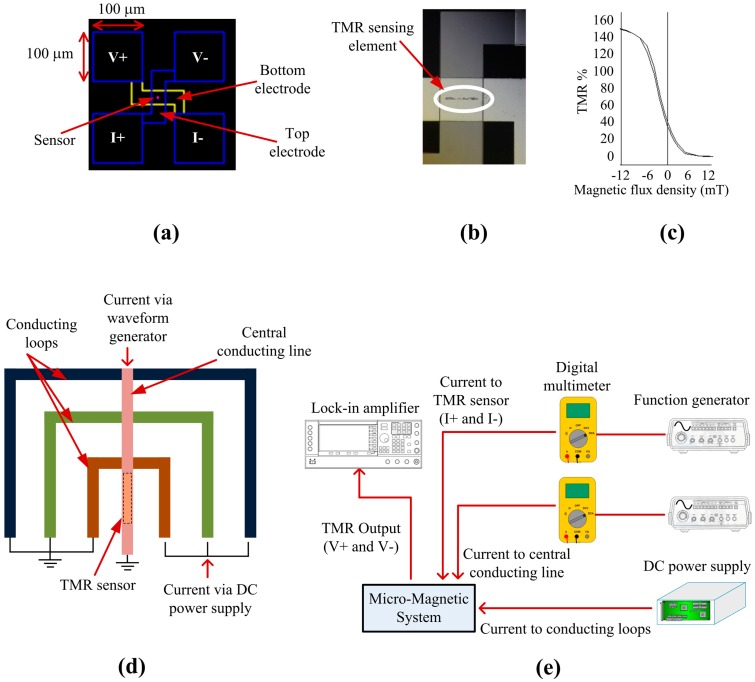
(**a**) Design of the TMR sensor; (**b**) Fabricated TMR sensing element; (**c**) Sensitivity of the TMR sensor; (**d**) MA and TMR sensor integration; (**e**) Experimental setup for magnetic bead detection.

The magnetoresistive sensor utilized was a tunneling magnetoresistive (TMR) sensor. The full TMR stack from top to bottom is: NiFe (16 nm)/Ta (0.21 nm)/Co40Fe40B20 (3.0 nm)/MgO (1.0 nm)/Co_40_Fe_40_B_20_ (1.64 nm)/Ta (0.21 nm)/Co_40_Fe_40_B_20_ (1.0 nm)/Ru (0.85 nm)/Co_70_Fe_30_ (2.0 nm)/IrMn (7.5 nm)/Ru (5 nm). The electrode configuration of the sensor is shown in Figure 3a, where V+ and V− are voltage contacts and I+ and I− are current contacts. The actual sensor element of 30 µm × 3 µm was sandwiched between the electrodes as shown in Figure 3b. Sensor characterization showed a resistance area product of 600 Ω·µm^2^ and a maximum TMR ratio of 150%. The sensitivity in the linear region between −5 to 5 mT is 10 %/mT (Figure 3c). To isolate the TMR sensor from the MA, a 200 nm layer of Si_3_N_4_ was deposited by a plasma-enhanced chemical vapor deposition (PECVD) system (PECVD Silicon Oxide and Nitride System, Oxford Instruments, Thornleigh, New South Wales, Australia). The parameters for the deposition procedure were: chamber pressure of 113 Pa, table temperature of 300 °C, 23 sccm SiH_4_, 20 sccm NH_3_, and 980 sccm N_2_. Reactive ion etching (RIE) was used to open the pads of the TMR sensor (Oxford Instruments Oxide/Nitride RIE and Strip), and finally, the MA was fabricated above the TMR sensor. The parameters for the etching procedure are: chamber pressure of 1.3 Pa, 90 sccm CHF_3_, and 10 sccm SF_6_.

The conducting loops of the MA and a central conducting line were deposited on top of the TMR sensor as shown in Figure 3d. The conducting loops of the MA have a common ground and the central conducting line has a separate ground. The current to the MA was supplied by a DC power supply and the current to the central conducting line was supplied by a waveform generator. Initially, the MA was activated and, once the initial set of SPBs reached the innermost loop, the current to the innermost loop was turned off and the current to the central conducting line was activated. Once SPBs moved to the central conducting line, the TMR sensor could detect the SPBs. The current to the central conducting line will remain on from this initial activation until all the required SPBs are attracted to the central conducting line by the MA. Once SPBs are trapped at the central conducting line they will remain there since the force exerted on them by the innermost conducting loop is insufficient to move them back from the central conducting line to the innermost conducting loop. A probe station equipped with a microscope (Probe Station, Cascade Microtech, Inc., Beaverton, OR, USA) was used to perform the magnetic bead detection experiments. As shown in Figure 3e, two probes (DCM 210 Series Precision Positioner, Cascade Microtech, Inc., Beaverton, OR, USA) were connected to a waveform generator (Agilent 33250A 80 MHz Function/Arbitrary Waveform Generator, Santa Clara, CA, USA) that provided an AC current of 200 µA at a frequency of 65 Hz to the I+ and I− pads of the TMR sensor, for its resistance measurement. A second waveform generator, connected to two more probes, was utilized to provide an AC current of 30 mA at a frequency of 331 Hz to the central conducting line to attract, trap and magnetize the SPBs for detection by the TMR sensor. In order to obtain the working frequency of the sensor we conducted an optimization test, where we found that the sensor response decreased for frequencies above several kHz and did not change significantly for frequencies below 1 kHz. Similarly, we found that the excitation frequency of 331 Hz applied to the central conducting line gave us a stable output. A DC power supply (Agilent E3646A, Santa Clara, CA, USA) was used to supply current to the conducting loops to transport SPBs to the central conducting line. Two digital multimeters (Agilent 34410A 6.5 Digital Multimeter, Santa Clara, CA, USA) were connected in series to each waveform generator to keep track of the current applied to the TMR sensor and the central conducting line. Two further probes were employed for the voltage measurement of the TMR sensor by a lock-in amplifier (SR850, Stanford Research Systems Inc., Sunnyvale, CA, USA) at the reference frequency of 396 Hz (331 Hz + 65 Hz) with a narrow bandwidth of 781 mHz. The sensor output due to the magnetic field of the conducting line was eliminated by using the signal when there were no SPBs on the TMR sensor, as a reference. 10 Dynabeads^®^ were dispensed to the surface of the micro-chip during experiments with the TMR sensor.

## 4. Results

### 4.1. Numerical Results

*V_mag_* of an SPB was calculated as follows with the aid of Table 1:

(5) $$M_{SPB}=\rho_{SPB}\times V_{SPB}$$

(6) $$M_{MC}=MC\times M_{SPB}$$

(7) $$V_{mag}=\frac{M_{MC}}{\rho_{\left({Fe}_{2}O_{3}/{Fe}_{3}O_{4}\right)}}$$

where *M_SPB_* (g) is the mass of the SPB, *ρ_SPB_* (g/m^3^) is the density of the SPB, *V_SPB_* (m^3^) is the volume of the SPB, *M_MC_* (g) is the mass of magnetic content, *MC* (%) is the magnetic content of the SPB and $\rho_{\left(\frac{{\text{Fe}}_{2}O_{3}}{{\text{Fe}}_{3}O_{4}}\right)}$ = 5.2 × 10^6^ (g/m^3^) is the average density of Fe_2_O_3_ and Fe_3_O_4_.

We define a variable, the coefficient of attraction (*C_att_*), which is the product of the volume susceptibility of an SPB and the magnetic volume of an SPB:

*C_att_* = *V_mag_* × *χ* (8)

**Figure 4:**
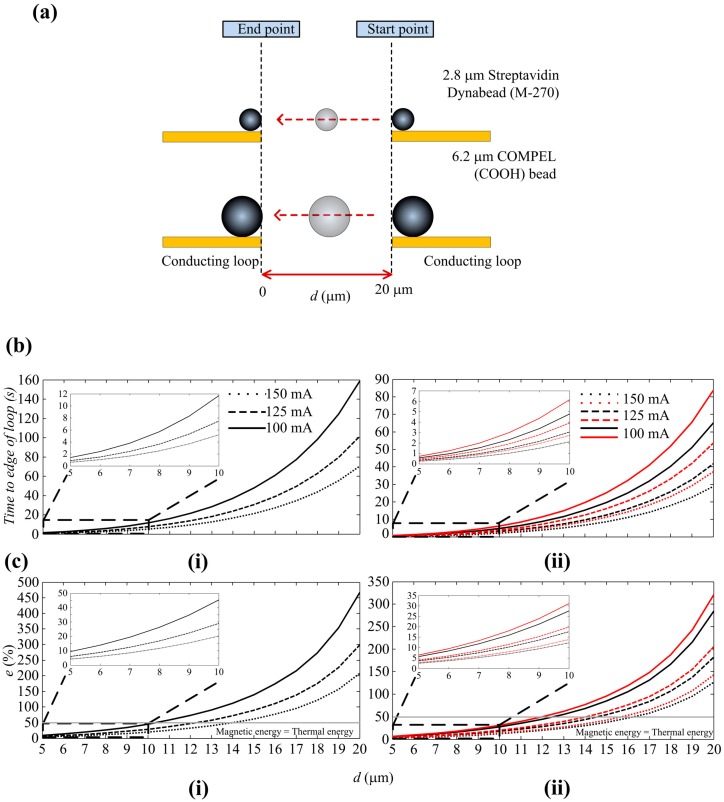
(**a**) Travel path of the SPBs; (**b**) The time taken for (i) COMPEL™ bead and (ii) Dynabead^®^ (black lines) and polystyrene YG particle tagged with a Dynabead^®^ (red lines) to travel to the edge of a loop, from a maximum distance of 20 μm from the edge; (**c**) Error caused by diffusion for (i) COMPEL™ bead and (ii) Dynabead^®^ and polystyrene YG particle tagged with a Dynabead^®^.

**Figure A3:**
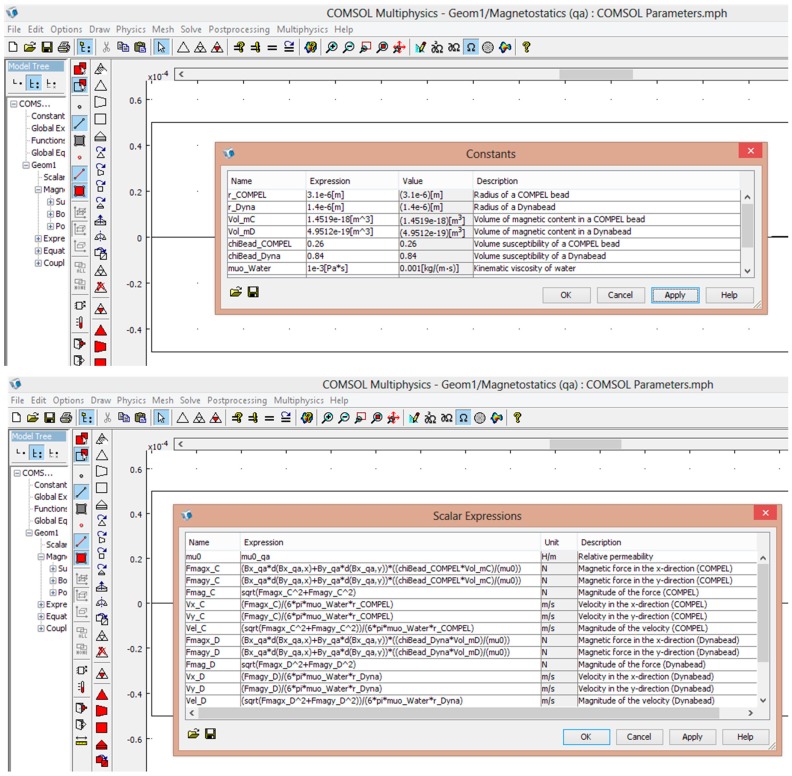
COMSOL constants and expressions for calculating magnetic force and velocity.

The COMSOL parameters for numerical analysis are shown in Figure A3. The travel path of the SPBs is shown in Figure 4a and Figure 4b(i,ii) show the time taken for a COMPEL^TM^ bead and a Dynabead^®^ to travel to an edge of a loop, from a maximum distance of 20 μm (*d* = 20 μm) from the edge of a loop, for *I* = 100, 125 and 150 mA. These plots only considered the *x*-direction of the magnetic force, since the movements of the SPBs are predominantly in the *x*-direction. The plots show that the time taken to reach the edge of the loop decreases when *I* increases, as expected. For example, the time taken for a COMPEL^TM^ bead to travel 20 μm is 158.9 s when *I* is 100 mA but reduces to 70.62 s when *I* is increased to 150 mA. Similarly, for a Dynabead^®^ the values are 65.15 s for *I* = 100 mA and 28.95 s when *I* = 150 mA. These results imply that the velocity at which an SPB can be attracted depends solely on *I* and *d*. However, during experiments, *I* cannot be increased, nor *d* decreased, infinitely. The corresponding current density for the maximum applied current of 150 mA is 3 × 10^10^ A/m^2^, which is well below the electromigration limit of 5.3 × 10^12^ A/m^2^ for Au. However, the heat transfer in the system should also be considered in the design of the MA. This not only depends on the current density but also the thermal conductivity of the substrate and the sample solution, as well as the experimental duration.

It can also be seen from Figure 4c(i,ii) that the closer the SPBs get to the loop, the faster they move towards it. *F_m,x_* decreases exponentially from the edge of the loop, therefore it takes an SPB much longer to travel a certain distance at the beginning of the migration process. Once the SPB is in the region of high gradients and flux magnitudes, it is quickly attracted to the surface of the loop. This is in agreement with Equation (1), which indicates that the higher the magnitudes and gradients of the field, the higher the force exerted on the SPB, and hence the higher the velocity of the SPB. For *I* = 125 mA, the times taken for a COMPEL™ bead to get trapped at the loop, when *d* = 20, 15, 10 and 5 μm, are 101.7, 30.59, 7.47 and 0.9 s, respectively. The corresponding values for a Dynabead^®^ are 41.7, 12.54, 3.06 and 0.36 s. It is interesting to note here that it takes longer for a COMPEL™ bead to travel a given distance than a Dynabead^®^. This can be explained by the characteristics of the two SPBs by referring to Table 1 and *C_att_*. The radius of a COMPEL™ bead is larger and therefore has a higher volume of magnetic material than a Dynabead^®^; generally, more nano-sized Fe_2_O_3_ and Fe_3_O_4_ can be packed into larger SPBs. However, the magnetic susceptibility of a COMPEL™ bead at magnetic flux densities below saturation is approximately 0.26, which is 3.23 times lower than the value for a Dynabead^®^. 

**Figure A4:**
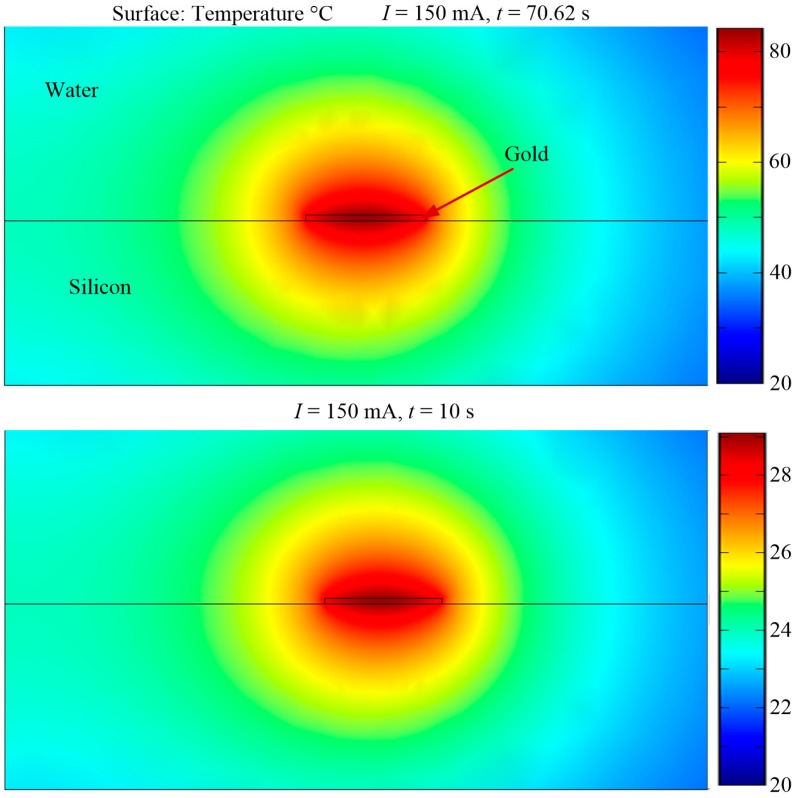

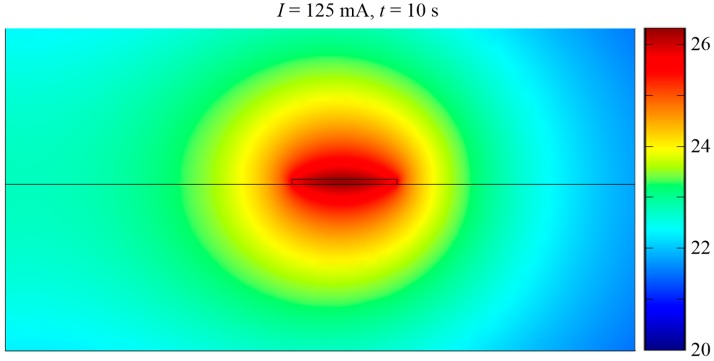
Heat transfer characteristics.

In comparison, a COMPEL™ bead has only 2.93 times more magnetic material than a Dynabead^®^. This results in the Dynabead^®^ having a larger *C_att_* than a COMPEL™ bead and therefore, being attracted to a magnetic source faster than a COMPEL™ bead. Moreover, the drag coefficient for a COMPEL™ bead is higher since the radius of a COMPEL™ bead is 2.2 times larger than a Dynabead^®^. Therefore, a Dynabead^®^ travels faster towards a loop than a COMPEL™ bead. The errors at 1 μm intervals were also calculated according to Equation (4). Several considerations were taken into account in choosing the time of 10 s for error calculations. Figure 4b(i) shows that a COMPEL™ bead takes 70.62 s to travel to a loop when *d* = 20 μm and *I* = 150 mA. However, this timeframe is not practical, since it corresponds to an increase in temperature by 60 °C from an initial temperature of 21 °C at the edge of the loop (See Figure A4 and Table A1). An increase in temperature of 60 °C did not have an influence on the COMPEL™ beads, Dynabeads^®^ and the polystyrene particles, which are all polymer based beads/particles and are stable up to a temperature of 100 °C, or the bond between the streptavidin, DNA and polystyrene particles, which generally require temperatures up to 90 °C and other additives to break the bond. However, it might damage other types of beads, cells attached to beads or the bond between beads and cells. The main issue we encountered in our experiments when the temperature increased was a reduction of the time it took for the droplet to evaporate. Experiments showed that the droplet formed on the surface of the MA when a sample solution was dispensed has a drying time of approximately 10 min at room temperature but decreased to 2 min when the temperature increased by 60 °C on the chip. This timeframe was not practical for observing and analyzing SPBs with the MA since, as the droplet started drying the SPBs and particles in the solution moved with the droplet, thus making control by magnetic forces impractical. The errors are shown for a COMPEL™ bead in Figure 4c(i) and a Dynabead^®^ in Figure 4c(ii). An error of 207% at *d* = 20 μm, as shown in Figure 4c(i), suggests that *d* = 20 μm is not feasible for a COMPEL™ bead. An error of 62% at *d* = 15 μm indicates that diffusion still dominates the magnetic force and therefore, a COMPEL™ bead will be less likely to move towards the edge of the loop. Generally, *e* >> 50% will ensure an SPB travels due to the magnetic force rather than diffusion. The error when *d* = 10 μm and *I* = 150 mA is 20% and the time taken to travel is 5.19 s. The error and time taken to travel when *I* = 125 mA do not vary too much from the corresponding values for 150 mA; the error is only 10% higher and the time taken is only 2 s longer. Furthermore, the temperature rise is also lower when *I* = 125 mA (see Figure A4). Based on the above analysis, *d* = 10 μm and *I* = 125 mA were chosen in the design of the MA. A characteristic measurement time of 10 s was considered appropriate given the time taken for a COMPEL™ bead to travel 10 μm is 7.47 s. Figure 4c(ii) shows that the error for a Dynabead^®^ taking into account these parameters is 18%, and increases to 20% when a 1 μm polymer bead is attached to it. Note that even though the errors and the time taken are lower when *d* = 5 μm, this value was considered to be too small since a COMPEL™ bead is 6 μm in diameter. Also, the times taken are too close to each other to observe the differences in travel velocities between a COMPEL™ and a Dynabead^®^.

The numerical analysis above assumed a dipole approximation to calculate ***F_m_*** on an SPB. However, since the sizes of the SPBs are comparable to the width of the Au loops as well as the distance between loops, we performed a numerical analysis by integrating the magnetic force over the volume of the SPB. By performing the integration we take into account that an SPB does not experience the same ***F_m_*** everywhere inside of its volume. We compared the main results with those obtained by a dipole approximation in Table 2. It can be seen that the times obtained by integration are longer than the times obtained by the dipole approximation. This is due to the fact that the side of an SPB closest to the magnetic field, as shown in Figure 4a, will experience a higher force than the side directly opposite to it, since the magnetic field will be lowest there. Therefore, unlike the dipole approximation, where the force is only taken at one point, the point closest to the magnetic field, the integration method takes into account the force within an SPB. The differences in times are greater for the larger COMPEL™ bead than the Dynabead^®^ since the COMPEL™ bead has a larger volume and will therefore have a larger variation of magnetic force inside of it. Both approximations, however, show that a Dynabead^®^ is faster than a COMPEL™ bead.

### 4.2. Experimental Results

**Figure 5:**
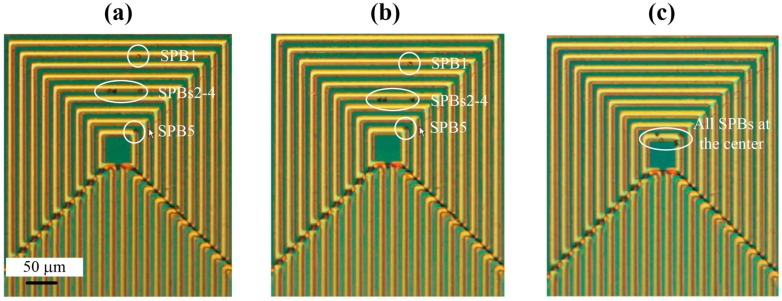
Experiments demonstrating the manipulation and transportation of COMPEL™ beads utilizing the magnetic actuator. (**a**) Five magnetic beads on the MA; (**b**) Movement of magnetic beads towards the center of the MA; (**c**) All magnetic beads manipulated to the center of the MA.

The capability of the MA to manipulate single SPBs with high precision is shown in Figure 5. Initially, as shown in Figure 5a, five COMPEL™ SPBs were trapped at different loops of the MA. There is one SPB on the innermost loop (SPB 5), three SPBs on the middle loop of loop set 2 (SPBs 2-4), and one SPB on the outermost loop of loop set 2 (SPB 1). In the next phase of the experiment, as shown in Figure 5b, the current is switched and the SPBs move one loop closer to the middle of the MA. Therefore, it can be deduced that the current had to be switched a further 6 times in order for the SPB in the outermost loop, SPB 1, to be manipulated to the innermost loop, and this can be seen in Figure 5c. The other three SPBs (SPBs 2–4) reached the innermost loop after the current was switched four times. All five SPBs remained trapped at the innermost loop until the current was switched off. Also, if the SPBs needed to be moved in the opposite direction, from the innermost loop towards the outermost loop, current could be switched between the loop sets in a reverse configuration. Similar experiments were also performed with bare Dynabeads^®^ and a polystyrene particle (polystyrene YG particle) attached to Dynabeads^®^. 

**Figure 6:**
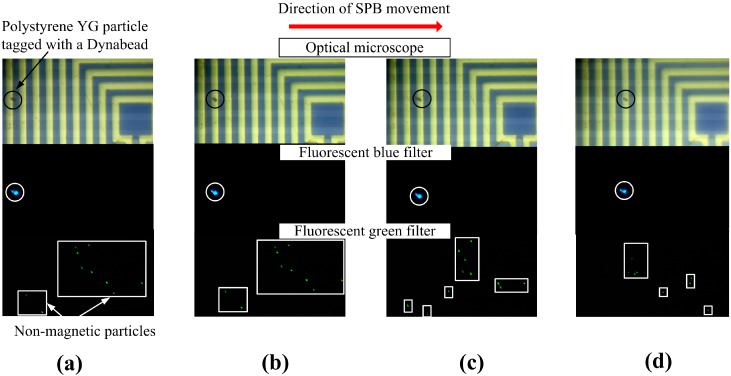
Transportation of a polystyrene YG particle tagged with a Dynabead^®^ in a heterogeneous solution (the tagged particle is circled while the unattached polystyrene PC particles are enclosed in squares/rectangles) from (**a**) the outermost loop of loop set 3 to (**b**) outermost loop of loop set 2 to (**c**) outermost loop of loop set 1 and (**d**) back to part of loop set 3.

Figure 6 shows the movement of a Dynabead^®^ with a polystyrene YG particle attached to it in a heterogeneous solution. One µL of sample solution containing both compound particles made of polystyrene YG particles attached to SPBs (fluorescent blue) and polystyrene PC particles (fluorescent green signal) was dispensed to the surface of the MA. The purpose of the polystyrene PC particles is merely to simulate a heterogeneous environment. Initially, when no current was applied to the MA, the SPBs and PC particles moved about randomly. When current was applied to loop set 1 a YG/Dynabead^®^ particle was attracted to the outermost ring and, thereby, separated from the non-magnetic particles. The area on the MA, where the YG particle is located, is magnified, to clearly show the separation procedure. Three images were taken for each of the manipulation steps shown, since the fluorescence microscope did not have the capability to observe all three colors simultaneously. Therefore, the fluorescence filter was set to match the spectral emission of the respective particles: the SPBs are black, the YG particles attached to the SPB are blue, and the unattached PC particles are green. The images on the top show only the SPB, the center shows only the YG particle attached to the SPB, and the bottom shows the PC particles. The PC particle was initially located at the outermost loop of set 3 (Figure 6a). When current was switched to loop set 2 this particle moved to the next loop, which is the outermost loop of loop set 2 (Figure 6b. The YG particles were not influenced by the action of the MA and, therefore, traveled in a random trajectory due to Brownian motion. When the current was switched to loop set 1, the YG particle followed the shift in the maximum flux density and was trapped at the outermost loop of loop set 1 (Figure 6c). Upon repetition of this process, the YG particle moved to the next ring, which is part of loop set 3 (Figure 6d). Finally, the YG particle reached the desired destination (center of the MA).

The average time taken for a COMPEL™ bead to move to the innermost loop from its adjacent loop was 8.1 ± 2.2 s. The average times taken for a Dynabead^®^ with and without a bound polymer were 4.1 ± 1.3 s and 6.3 ± 1.8 s, respectively. The time obtained for COMPEL™ beads are closer to the numerical analysis results obtained using a dipole approximation while the time for Dynabeads^®^ are closer to the numerical results obtained by volume integration. These results can be explained by considering the magnetite distribution inside of the SPBs. Dynabeads^®^ have a uniform dispersion of magnetite nanocrystals inside of a polymer matrix, whereas COMPEL™ beads have the magnetite crystals deposited very near to the surface. These types of distributions are commonly known as ‘Fruitcake’ and ‘Orange Peel’, respectively [58]. When performing the volume integration we assumed the magnetite is distributed uniformly inside of the SPB, but in reality this is only true for Dynabeads^®^ since the COMPEL™ beads will have a higher susceptibility near the edge of the beads and little or no magnetic content in the middle region. As shown in Figure 4a, we calculated the times for the edge of an SPB to move from one loop to another. In the dipole approximation force on the SPB is taken as a point, which is akin to the edge of the SPB, whereas in the volume integration the whole volume of the SPB is considered. Since the COMPEL™ beads have a higher magnetic content at the edge, the experimental results are closer to the dipole approximation results while the experimental results for the Dynabeads^®^ are closer to the volume integration results because the magnetic content inside the bead is uniform.

The mean time is higher for the COMPEL™ beads than the time predicted by numerical analysis using the dipole approximation method while for the Dynabeads^®^ it is lower than the time obtained by numerical analysis using the volume integration method. The most plausible reason for experimental times to be higher than the numerical analysis is the adhesion of SPBs to the surface of the substrate, which can influence the manipulation of the SPBs due to friction. COMPEL™ beads are larger than Dynabeads^®^ so friction and adhesion will have a larger effect. Even though the chips were treated before experiments, and a noticeable enhancement of the transportation of SPBs was observed, it was not possible to fully eliminate friction and adhesion The treatment procedure involved the chip being treated with 10 mg/mL bovine serum albumin (BSA) and 0.1% (*v*/*v*) sodium dodecyl sulfate (SDS) solution for 15 min. BSA is known to inhibit cell adhesion and SDS prevents binding of SPBs or undesired waste to the substrate of the chip and the Au wires. Also, it removed dead cells, particles and SPBs left from previous experiments. Finally, the chip was washed for 15 min with 0.2% (*v*/*v*) of Tween 20, which is a non-ionic, gentle surfactant that prevents specific adsorption and has long term stability. The movement of the SPBs can also be hindered if the surface of the substrate is not entirely uniform, and variations in diameter and susceptibility values of the SPBs as well as the distribution of the nanocrystals in the polymer matrix contribute to the differences between the times of travel obtained by numerical analysis and experimental results.

**Figure 7:**
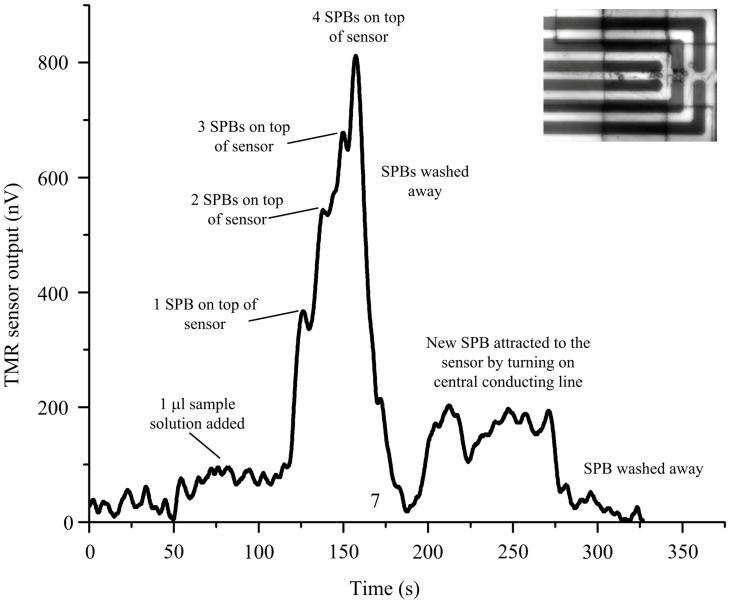
Manipulation and detection of magnetic beads by the micro-chip.

Finally, we demonstrate how the simple, yet effective, design of the MA can be integrated with a tunnel magnetoresistive (TMR) sensor to form an integrated micro-chip for the detection of SPBs. Figure 7 shows the output signal of the TMR sensor after dispensing 1 µL of the sample solution. Initially we used the MA to attract SPBs to the innermost loop in the MA, Then, after 25 s, the central conducting line was turned on, and the MA was turned off, to attract the SPBs from the innermost loop of the MA to the central conducting line and, hence, to the top of the TMR sensor. As the first SPB reached the top of the sensor after 125 s, a signal of about 250 nV was generated. Every additional SPB that reached the sensor surface increased the signal, as can be seen at 140 s, 150 and 165 s. After washing away the SPBs, the signal dropped to zero, at 175 s. By turning on the current at the central conducting line, a new bead was attracted (250 s) before washing it off again (300 s). Individual SPBs were detected with a signal to noise ratio of about 3, which, in combination with the MA, provides a powerful tool for bioanalytical experiments.

Even though individual SPBs can be detected by the TMR sensor, the results show that the increase in the sensor output decreases with each attracted SPB. The magnitude of the magnetic stray field of the SPB experienced by the sensor also depends on the position of the SPB relative to the sensor [59]. Moreover, the magnetic field magnetizing the SPB is not homogenous all along the trapping area and changes with position. It is important to note here that the proposed sensing system is sensitive enough to detect the stray field from an individual bead, but the signal cannot reveal the exact number of SPBs when multiple SPBs are present on top of the sensor. However, if the number of SPBs is large and they can be functionalized on top of, or trapped in a well that covers, the area of the sensor, the number of SPBs can be evaluated since we know the area on top of the sensor and the maximum number of SPBs that can be trapped on/in it.

## 5. Discussion

In this work, we presented an effective way to precisely control the movement of single SPBs by utilizing a magnetic actuator. The design of the MA is based on square-shaped, conducting loop sets that are employed to produce magnetic flux gradients, which, in turn, exert a force on SPBs. We showed that taking into account the iron oxide/magnetite content rather than the volume of the whole SPB represents a more accurate method of analyzing forces on an SPB and also considered the effect of diffusion on the distance an SPB can be attracted from.

Simulations based on FEM were carried out on a 2-D model, to help understand the manipulation of SPBs with the MA and the resulting SPB trajectories in a liquid medium. The results showed that a current of 125 mA produces a maximum magnetic flux density of 10 mT at the edge of Au loops with a cross-sectional area of 10 µm × 0.5 µm. A higher current could generate larger magnetic forces, resulting in faster SPB velocities, but it is limited by Joule heating. For example, heat transfer analysis showed that a current of 125 mA applied for 10 s increases the temperature by 5 °C. Magnetic forces were calculated for two types of SPBs, 6.2 µm COMPEL™ beads and 2.8 µm Dynabeads^®^. The COMPEL™ beads have 2.93 times the magnetic content of Dynabeads^®^ but their susceptibility is 3.23 times lower. Therefore, even though Dynabeads^®^ are smaller in size than COMPEL™ beads they experience a larger magnetic force and, hence, travel faster towards a magnetic source. Numerical analysis showed that a Dynabead^®^ travelled to the surface of a conducting loop 2.4 times faster than a COMPEL™ bead when it was initially placed 10 µm from the conducting loop. Experimental results showed a ratio of 2.0, confirming the numerical analysis, the difference being related to effects that were neglected in the model, such as friction between SPB and substrate surface. Binding a polystyrene particle to a Dynabead^®^ influences the speed of travel. The ratios of the velocities of a COMPEL™ bead to a polystyrene particle tagged with a Dynabead^®^ obtained by numerical and experimental analysis reduced to 1.9 and 1.3, respectively. This opens up possibility of separating molecules/cells according to size. The critical boundary, where the magnetic and thermal energies are balanced and the error caused by diffusion is 50%, is 12.3 µm for a COMPEL™ bead, 14.62 µm for a Dynabead^®^ and 14.0 µm for the polystyrene particle tagged with a Dynabead^®^. This critical boundary can be expanded if the current is increased and/or if the experimentation time is increased, but care has to be taken with respect to the heat generated in the system. We also compared the numerical results, which took into account a dipole approximation, to analysis performed by integrating the force over the volume of an SPB. The results for the time taken for a SPB to travel 5, 10, 15 and 20 µm towards an Au wire revealed that the difference between the two methods is rather small and more relevant for the larger COMPEL™ bead than the smaller Dynabead^®^. This is to be expected, as the dipole approximation method assumes an infinitely small point, and therefore, as the SPB gets smaller the approximation using the volume integral method will also get closer to the result obtained by the dipole approximation method. The experimental results showed that the time taken for a Dynabead^®^ to travel 10 µm agrees more with the volume integration method and the time taken for a COMPEL™ bead to travel the same distance agrees more with the dipole approximation method. We believe the magnetite distribution of the two SPBs contributed to these results; the Dynabead^®^ has a uniform magnetite distribution, which is in line with the assumption that the susceptibility is uniform in the whole bead, and the COMPEL™ bead has magnetite distributed closer to the edge of the bead, which is more in line with the assumption that the edge of the bead has a higher susceptibility than inner regions of the bead.

## 6. Conclusions

A magnetic actuator (MA) was fabricated on a Si/SiO_2_ substrate in combination with a magnetoresistive (MR) sensor for the on-chip detection of SPBs. Experimental results demonstrated the possibility of accurate manipulation and movement of SPBs, as well as the concentration of SPBs to a target site at the center of the MA where the TMR sensor was located. The actuator concept can easily be extended over a large area to manipulate SPBs simply by electrical signals over large distances or for increased concentration efficiency. A numerical method has been presented that enables calculating the SPBs’ response to the operation of the MA. The simulation results were in good agreement with the experimental ones and thus can be used to optimize design parameters for specific applications. The TMR sensor was able to detect the SPBs that were manipulated to its location by the MA. Such a micro-chip system is capable of both manipulation and detection of either an individual SPB or a large number of them.

## Figures and Tables

**Table 1:** Superparamagnetic bead characteristics [56,57].

Bead Type	Radius (m) × 10^−6^	Volume (m^3^) × 10^−16^	Density (g/m^3^) × 10^6^	Mass (g) × 10^−10^	Magnetic Content (%)	Mass of Magnetic Content (g) × 10^−12^	Volume of Magnetic Content (m^3^) × 10^−18^	Volume Susceptibility
COMPEL^TM^	3.1	1.2479	1.1	1.3727	5.5	7.5499	1.4519	0.26
Dynabead^®^	1.4	0.11494	1.6	0.1839	14	2.5746	0.49512	0.84

**Table 2:** Comparison of the dipole approximation method to the volume integration method with respect to time taken for an SPB to travel to an edge of a loop from distances of 5, 10, 15 and 20 μm.

Bead Type	Method	5 μm	10 μm	15 μm	20 μm
COMPEL™	Dipole approximation	0.9 s	7.47	30.59	101.7
	Volume integration	3.9	18.7	60.6	164.8
Dynabead^®^	Dipole approximation	0.36	3.06	12.54	41.7
	Volume integration	0.75	4.72	17.3	55.79

## Appendix A

**Table A1:** COMSOL heat transfer parameters.

Material	Thermal Conductivity (W/m·K)	Density	Material	Thermal conductivity (W/m·K)
Water	130	2329	700	-
Silicon	0.58	1000	4183	-
Gold	317	19300	129	*Q* × *t*

## References

[1] Pankhurst Q.A., Connolly J., Jones S.K., Dobson J. (2003). Applications of magnetic nanoparticles in biomedicine. J. Phys. D: Appl. Phys..

[2] Ehresmann A., Koch I., Holzinger D. (2015). Manipulation of Superparamagnetic Beads on Patterned Exchange-Bias Layer Systems for Biosensing Applications. Sensors.

[3] Gijs M.A.M., Lacharme F., Lehmann U. (2010). Microfluidic applications of magnetic particles for biological analysis and catalysis. Chem. Rev..

[4] Jiang Y., Wang H., Li S., Wen W. (2014). Applications of Micro/Nanoparticles in Microfluidic Sensors: A Review. Sensors.

[5] Yassine O., Gooneratne C.P., Smara D.A., Li F., Mohammed H., Merzaban J., Kosel J. (2014). Isolation of cells for selective treatment and analysis using a magnetic microfluidic chip. Biomicrofluidics.

[6] Sakaki K., Foulds I.G., Liu W., Dechev N., Burke R.D., Park E.J. (2009). RoboSCell: An automated single cell arraying and analysis instrument. Biomed. Microdevices.

[7] Liu W., Dechev N., Foulds I.G., Burke R., Parameswaran A., Park E.J. (2009). A novel permalloy based magnetic single cell micro array. Lab Chip.

[8] Li F., Kosel J. (2014). An efficient biosensor made of an electromagnetic trap and a magneto-resistive sensor. Biosens. Bioelectron..

[9] Sofla A., Cirkovic B., Hsieh A., Miklas J.W., Filipovic N., Radisic M. (2013). Enrichment of live unlabelled cardiomyocytes from heterogeneous cell populations using manipulation of cell settling velocity by magnetic field. Biomicrofluidics.

[10] Gooneratne C.P., Yassine O., Giouroudi I., Kosel J. (2013). Selective Manipulation of Superparamagnetic Beads by a Magnetic Microchip. IEEE Trans. Magn..

[11] Darabi J., Guo C. (2013). On-chip magnetophoretic isolation of CD4 + T cells from blood. Biomicrofluidics.

[12] Tarn M.D., Peyman S.A., Pamme N. (2013). Simultaneous trapping of magnetic and diamagnetic particle plugs for separations and bioassays. RSC Adv..

[13] Pamme N. (2006). Magnetism and Microfluidics. Lab Chip.

[14] Liu C., Stakenborg T., Peeters S., Lagae L. (2009). Cell manipulation with magnetic particles toward microfluidic cytometry. J. Appl. Phys..

[15] Suwa M., Watarai H. (2011). Magnetoanalysis of micro/nanoparticles: A review. Anal. Chim. Acta.

[16] Donolato M., Vavassori P., Gobbi M., Deryabina M., Hansen M.F., Metlushko V., Ilic B., Cantoni M., Petti D., Brivio S. (2010). On-Chip Manipulation of Protein-Coated Magnetic Beads via Domain-Wall Conduits. Adv. Mater..

[17] Anandakumar S., Rani V.S., Oh S., Sinha B.L., Takahashi M., Kim C. (2010). Translocation of bio-functionalized magnetic beads using smart magnetophoresis. Biosens. Bioelectron..

[18] Rapoport E., Montana D., Beach G.S.D. (2012). Integrated capture, transport, and magneto-mechanical resonant sensing of superparamagnetic microbeads using magnetic domain walls. Lab Chip.

[19] Sushruth M., Ding J., Duczynski J., Woodward R.C., Begley R., Fanghor H., Fuller R.O., Adeyeye A.O., Kostylev M., Metaxas P.J. (2016). Resonance-based Detection of Magnetic Nanoparticles and Microbeads Using Nanopatterned Ferromagnets.

[20] Corte-León H., Krzysteczko P., Schumacher H.W., Manzin A., Cox D., Antonov V., Kazakova O. (2015). Magnetic bead detection using domain wall-based nanosensor. J. Appl. Phys..

[21] Quynh L.K., Tu B.D., Dang D.X., Viet D.Q., Hien L.T., Huong Giang D.T., Duc N.H. (2016). Detection of magnetic nanoparticles using simple AMR sensors in Wheatstone bridge. J. Sci. Adv. Mat. Dev..

[22] Monticelli M., Torti A., Cantoni M., Petti D., Albisetti E., Manzin A., Guerriero E., Sordan R., Gervasoni G., Carminati M. (2016). On-Chip Magnetic Platform for Single-Particle Manipulation with Integrated Electrical Feedback. Small.

[23] Gooneratne C.P., Kurnicki A., Yamada S., Mukhopadhyay S.C., Kosel J. (2013). Analysis of the Distribution of Magnetic Fluid inside Tumors by a Giant Magnetoresistance Probe. PLoS ONE.

[24] Freitas P.P., Ferreira H.A., Graham D.L., Clarke L.A., Amaral M.D., Martins V., Fonseca L., Cabral J.S., Johnson M. (2004). Magnetoelectronics.

[25] Albon C., Weddemann A., Augel A., Rott K., Hütten A. (2009). Tunneling magnetoresistance sensors for high resolutive particle detection. Appl. Phys. Lett..

[26] Cubells-Beltran M.D., Reig C., De Marcellis A., Figueras E., Yufera A., Zadov B., Paperno E., Cardoso S., Freitas P.P. (2014). Monolithic integration of Giant Magnetoresistance (GMR) devices onto standard processed CMOS dies. Microelectron. J..

[27] Wang S.X., Li G. (2008). Advances in Giant Magnetoresistance Biosensors with Magnetic Nanoparticle Tags: Review and Outlook. IEEE. Trans. Magn..

[28] Cubells-Beltrán M.D., Reig C., Madrenas J., De Marcellis A., Santos J., Cardoso S., Freitas P.P. (2016). Integration of GMR Sensors with Different Technologies. Sensors.

[29] Gaster R.S., Xu L., Han S.J., Wilson R.J., Hall D.A., Osterfeld S.J., Yu H., Wang S.X. (2011). Quantification of protein interactions and solution transport using high-density GMR sensor arrays. Nat. Nanotechnol..

[30] Osterfeld S.J., Yu H., Gaster R.S., Caramuta S., Xu L., Han S.J., Hall D.A., Wilson R.J., Sun S., White R.L. (2008). Multiplex protein assays based on real-time magnetic nanotag sensing. Proc. Natl. Acad. Sci. USA.

[31] Graham D.L., Ferreira H.A., Feliciano N., Freitas P.P., Clarke L.A., Amaral M.D. (2005). Magnetic field-assisted DNA hybridisation and simultaneous detection using micron-sized spin-valve sensors and magnetic nanoparticles. Sens. Actuators B.

[32] Ferreira H.A., Graham D.L., Feliciano N., Clarke L.A., Amaral M.D., Freitas P.P. (2005). Detection of cystic fibrosis related DNA targets using AC field focusing of magnetic labels and spin-valve sensors. IEEE. Trans. Magn..

[33] Rizzi G., Østerberg F.W., Dufva M., Hansen M.F. (2014). Magnetoresistive sensor for real-time single nucleotidepolymorphism genotyping. Biosens. Bioelectron..

[34] Koets M., van der Wijk T., van Eemeren J.T.W.M., van Amerongen A., Prins M.W.J. (2009). Rapid DNA multi-analyte immunoassay on a magneto-resistance biosensor. Biosens. Bioelectron..

[35] Mak A.C., Osterfeld S.J., Yu H., Wang S.X., Davis R.W., Jejelowo O.A., Pourmand N. (2009). Sensitive giant magnetoresistive-based immunoassay for multiplex mycotoxin detection. Biosens. Bioelectron..

[36] Kokkinis G., Cardoso S.F., Cardoso F.A., Giouroudi I. (2014). Microfluidics for the Rapid Detection of Pathogens Using Giant Magnetoresistance Sensors. IEEE Trans. Magn..

[37] Kim D., Lee J.R., Shen E., Wang S.X. (2013). Modeling and experiments of magneto-nanosensors for diagnostics of radiation exposure and cancer. Biomed. Microdevices.

[38] Loureiro J., Andrade P.Z., Cardoso S., da Silva C.L., Cabral J.M., Freitas P.P. (2011). Magnetoresistive chip cytometer. Lab Chip.

[39] Lei Z.Q., Li L., Li G.J., Leung C.W., Shi J., Wong C.M., Lo K.C., Chan W.K., Mak C.S.K., Chan S.B. (2012). Liver cancer immunoassay with magnetic nanoparticles and MgO-based magnetic tunnel junction sensors. J. Appl. Phys..

[40] Gaster R.S., Hall D.A., Wang S.X. (2011). nanoLAB: An ultraportable, handheld diagnostic laboratory for global health. Lab Chip.

[41] Lian J., Chen S., Qiu Y., Zhang S., Shi S., Gao Y. (2012). A fully automated in vitro diagnostic system based on magnetic tunnel junction arrays and superparamagnetic particles. J. Appl. Phys..

[42] Schotter J., Shoshi A., Brueckl H. (2009). Development of a magnetic lab-on-a-chip for point-of-care sepsis diagnosis. J. Magn. Magn. Mater..

[43] Weddemann A., Albon C., Auge A., Wittbracht F., Hedwig P., Akemeier D., Rott K., Meißner D., Jutzi P., Hütten A. (2010). How to design magneto-based total analysis systems for biomedical applications. Biosens. Bioelectron..

[44] Lagae L., Wirix-Speetjens R., Das J., Graham D., Ferreira H., Freitas P.P.F., Borghs G., De Boeck J. (2002). On-chip manipulation and magnetization assessment of magnetic bead ensembles by integrated spin-valve sensors. J. Appl. Phys..

[45] Wirix-Speetjens R., Fyen W., De Boeck J., Borghs G. (2006). Single magnetic particle detection: Experimental verification of simulated behavior. J. Appl. Phys..

[46] Wirix-Speetjens R., Reekmans G., De Palma R., Liu C., Laureyn W., Borghs G. (2007). Magnetoresistive biosensors based on active guiding of magnetic particles towards the sensing zone. Sens. Actuators B..

[47] Megens M., Prins M. (2005). Magnetic biochips: A new option for sensitive diagnostics. J. Magn. Magn. Mater..

[48] Van Ommering K., Lamers C.C.H., Nieuwenhuis J.H., van IJzendoorn L.J., Prins M.W.J. (2009). Analysis of individual magnetic particle motion near a chip surface. J. Appl. Phys..

[49] Gooneratne C.P., Giouroudi I., Liang C., Kosel J. (2011). A giant magnetoresistance ring-sensor based microsystem for magnetic bead manipulation and detection. J. Appl. Phys..

[50] Gooneratne C.P., Liang C., Kosel J. (2011). A planar conducting microstructure to guide and confine magnetic beads to a sensing zone. Microelectron. Eng..

[51] Gooneratne C.P., Giouroudi I., Kosel J. (2011). A planar conducting micro-loop structure for transportation of magnetic beads: An approach towards rapid sensing and quantification of biological entities. Sens. Lett..

[52] Donolato M., Dalslet B.T., Hansen M.K. (2012). Microstripes for transport and separation of magnetic particles. Biomicrofluidics.

[53] Kokkinis G., Keplinger F., Giouroudi I. (2013). On-chip microfluidic biosensor using superparamagnetic microparticles. Biomicrofluidics.

[54] Gooneratne C.P., Liang C., Giouroudi I., Kosel J. (2012). An integrated micro-chip for rapid detection of magnetic particles. J. Appl. Phys..

[55] Li F., Kodzius R., Gooneratne C.P., Foulds I., Kosel J. (2014). Magneto-mechanical trapping systems for biological target detection. Microchim. Acta.

[56] Fonnum G., Johansson C., Molteberg A., Mørup S., Aksnes E. (2005). Characterisation of Dynabeads^®^ by magnetization measurements and Mössbauer spectroscopy. J. Magn. Magn. Mater..

[57] Bangs Laboratories, Inc.™. https://www.bangslabs.com.

[58] Ruffert C. (2016). Magnetic Bead—Magic Bullet. Micromachines.

[59] Henriksen A.D., Ley M.W.H., Flyvbjerg H., Hansen M.F. (2015). Configurational Statistics of Magnetic Bead Detection with Magnetoresistive Sensors. PLoS ONE.

